# Structural and Functional Divergence of Gonadotropin-Inhibitory Hormone from Jawless Fish to Mammals

**DOI:** 10.3389/fendo.2014.00177

**Published:** 2014-10-24

**Authors:** Satoshi Ogawa, Ishwar S. Parhar

**Affiliations:** ^1^Brain Research Institute, Jeffrey Cheah School of Medicine and Health Sciences, Monash University Malaysia, Petaling Jaya, Malaysia

**Keywords:** LPXRFa, GnRH, reproduction, teleosts, gonadotropin

## Abstract

Gonadotropin-inhibitory hormone (GnIH) was discovered as a novel hypothalamic peptide that inhibits gonadotropin release in the quail. The presence of GnIH-homologous peptides and its receptors (GnIHRs) have been demonstrated in various vertebrate species including teleosts, suggesting that the GnIH-GnIHR family is evolutionarily conserved. In avian and mammalian brain, GnIH neurons are localized in the hypothalamic nuclei and their neural projections are widely distributed. GnIH acts on the pituitary and gonadotropin-releasing hormone neurons to inhibit reproductive functions by decreasing gonadotropin release and synthesis. In addition, GnIH-GnIHR signaling is regulated by various factors, such as environmental cues and stress. However, the function of fish GnIH orthologs remains inconclusive because the physiological properties of fish GnIH peptides are debatable. This review summarizes the current research progress in GnIH-GnIHR signaling and their physiological functions in vertebrates with special emphasis on non-mammalian vertebrate species.

## Introduction: Discovery of GnIH

When the reproductive axis is triggered, gonadotropin-releasing hormone (GnRH), a neuropeptide involved in regulating vertebrate reproduction, is released from the hypothalamus. The released GnRH then enters into the anterior pituitary gland and triggers the release of gonadotropins: luteinizing hormone (LH) and follicle-stimulating hormone (FSH) (1, 2). These gonadotropins act on the gonads to stimulate the synthesis and release of gonadal steroids (3). Kisspeptin, the peptide product of *KISS1*/*Kiss1* gene and its cognate receptor (GPR54 = kisspeptin receptor) has been well recognized as a potent regulator of GnRH release in vertebrates (4, 5). In mammals, kisspeptin immunoreactive fibers are seen in close apposition with GnRH neurons (6, 7) and with GnRH axons in the median eminence (ME) in the primates (8). Furthermore, GPR54 expression has been demonstrated in GnRH neurons from a non-mammalian species, the cichlid fish, tilapia (9), suggesting that kisspeptin plays stimulatory role via its action on GnRH neurons. In 2000, Tsutsui and his colleagues discovered a novel hypothalamic neuropeptide, termed gonadotropin-inhibitory hormone (GnIH) in the Japanese quail, *Coturnix japonica* that directly acts on the pituitary gland, thus impeding gonadotropin release (10). This was the first illustration of a hypothalamic neuropeptide demonstrating inhibitory effects on reproduction in any vertebrate (10).

## Structure of GnIH and GnIH Receptor Orthologs in Vertebrates

### GnIH and GnIH orthologs

**Figure 1:**
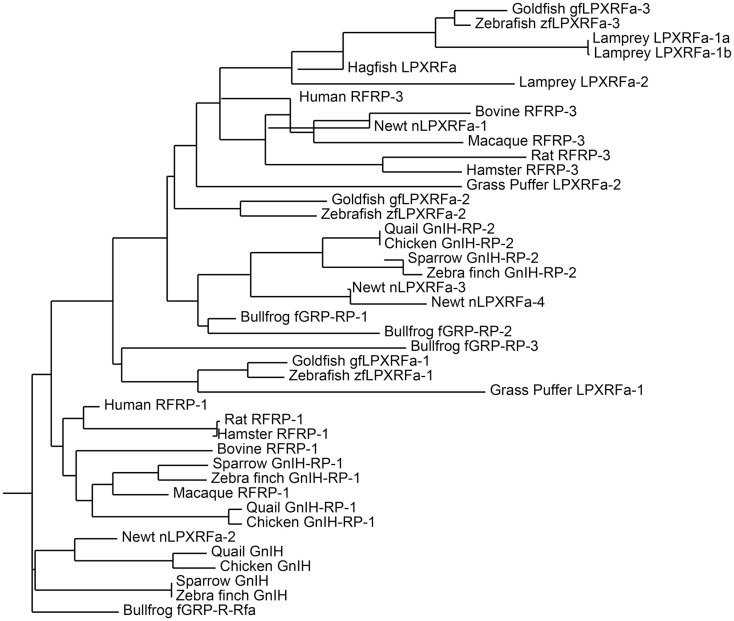
**Phylogenetic tree of GnIH and its homologous peptides sequences in vertebrates**. The phylogenetic tree was constructed by MEGA 3.1 using the neighbor-joining method. The amino acid sequences analyzed for the phylogenetic tree construction are listed in Table 1.

GnIH belongs to the RFamide family of peptides as it contains RFamide motifs (Arg-Phe-NH_2_) at its C-terminus. The amino acid sequence of GnIH and its orthologs in various vertebrates and their phylogenetic relationship are demonstrated in Table 1 and Figure 1.

**Table 1:** **Comparison of amino acid sequences of GnIH and its homologous peptides from jawless fish to mammals**.

Animal	Species	Name	Amino acid sequence	Distribution (mRNA or peptides)^a^	Mode of action	Reference
**JAWLESS FISH**						
Sea lamprey	*Petromyzon marinus*	LPXRFa-1a	SGVGQGRSSKTLF**QPQRFa**	B, T, O		(11)
		LPXRFa-1b	AALRSGVGQGRSSKTLF**QPQRFa**			(11)
		LPXRFa-2	SEPFWHRT**RPQRFa**			(11)
Hagfish	*Myxine glutinosa*	LPXRFa	A**LPQRFa**			(12)
**JAWED FISH**						
Goldfish	*Carassius auratus*	gfLPXRFa-1	PTHLHAN**LPLRFa**	B		(13)
		gfLPXRFa-2	AKSNIN**LPQRFa**			(13)
		gfLPXRFa-3	SGTGLSAT**LPQRFa**			(13)
Zebrafish	*Danio rerio*	zfLPXRFa-1	PAHLHAN**LPLRFa**	B, E, T, O, M, K, SP, G		(14)
		zfLPXRFa-2	APKSTIN**LPQRFa**			(14)
		zfLPXRFa-3	SGTGPSAT**LPQRFa**			(14)
Grass Puffer	*Takifugu niphobles*	LPXRFa-1	SLDMERINIQVSPTSGKVSLP	B, P, E, K, SP		(15)
			TIVRLYPPTLQPHHQHVN**MPMRFa**	
		LPXRFa-2	DGVQGGDHVPNLNPN**MPQRFa**			(15)
Nile tilapia	*Oreochromis niloticus*	LPXRFa-1	Ac-TLLSSNDGTYSVRKQPHQETKNEIHRSLDL	B, P, T, O		(14, 16)
			ESFNIRVAPTTSKFSLPTIIRFYPPTVKPLHLHAN**MPLRFa**	
		LPXRFa-2	p-QSDERTPNSSPN**LPQRFa**			(14, 16)
		LPXRFa-3	Ac-APNQL**LSQRF**E			(14, 16)
**AMPHIBIAN**						
Bullfrog	*Rana catesbeiana*	fGRP/R-Rfa	SLKPAAN**LPLRFa**	B		(17, 18)
		fGRP-RP-1	SIPN**LPQRFa**			(19)
		fGRP-RP-2	YLSGKTKVQSMAN**LPQRFa**			(19)
		fGRP-RP-3	AQYTNHFVHSLDT**LPLRFa**			(19)
European green frog	*Rana esculenta*	R-RFa	SLKPAAN**LPLRFa**	B		(20)
Japanese red-bellied newt	*Cynops pyrrhogaster*	nLPXRFa-1	SVPN**LPQRFa**	B		(21)
		nLPXRFa-2	MPHASAN**LPLRFa**			(21)
		nLPXRFa-3	SIQPLAN**LPQRFa**			(21)
		nLPXRFa-4	APSAGQFIQTLAN**LPQRFa**			(21)
**BIRD**						
Japanese Quail	*Coturnix japonica*	GnIH	SIKPSAY**LPLRFa**	B, T, O	GnRH1	(10, 22, 23)
		GnIH-RP-1	SLNFEEMKDWGSKNFMKVNTPTVNKVPNSVAN**LPLRFa**			(24)
		GnIH-RP-2	SSIQSLLN**LPQRFa**			(24)
Chicken	*Gallus gallus*	GnIH	SIRPSAY**LPLRFa**	B		(25)
		GnIH-RP-1	SLNFEEMKDWGSKNFLKVNTPTVNKVPNSVAN**LPLRFa**			(25)
		GnIH-RP-2	SSIQSLLN**LPQRFa**			(25)
Gambel’s white-crowned sparrow	*Zonotrichia leucophrys gambelii*	GnIH	SIKPFSN**LPLRFa**	B	GnRH2	(26, 27)
		GnIH-RP-1	SLNFEEMEDWGSKDIIKMNPFTASKMPNSVAN**LPLRFa**			(26)
		GnIH-RP-2	SPLVKGSSQSLLN**LPQRFa**			(26)
European starling	*Sturnus vulgaris*	GnIH	SIKPFAN**LPLRFa**	B, T, O	GnRH1, GnRH2	(28)
		GnIH-RP-1	SLNFDEMEDWGSKDIIKMNPFTVSKMPNSVAN**LPLRFa**			(28)
		GnIH-RP-2	GSSQSLLN**LPQRFa**			(28)
Zebra finch	*Taeniopygia guttata*	GnIH	SIKPFSN**LPLRFa**	B	GnRH1	(29)
		GnIH-RP-1	SLNFEEMEDWRSKDIIKMNPFAASKMPNSVAN**LPLRFa**			(29)
		GnIH-RP-2	SPLVKGSSQSLLN**LPQRFa**			(29)
**MAMMAL**						
Human being	*Homo sapiens*	RFRP-1	MPHSFAN**LPLRFa**	B		(30)
		RFRP-3	VPN**LPQRFa**	B	GnRH1	(30)
Rhesus macaque	*Macaca mulatta*	RFRP-1	MPHSVTN**LPLRFa**	B		(31)
		RFRP-3	SGRNMEVSLVRQVLN**LPQRFa**	B	GnRH1, GnRH2, dopamine, β-endorphin	(31–33)
Mouse	*Mus musculus*	RFRP-1	SVSFQELKDWGAKKVIKMSPAPANKVPHSAAN**LPLRFa**	B		(34)
		RFRP-3	ANMEAGTRSHFPS**LPQRFa**	B	GnRH1, kisspeptin	(34, 35)
Rat	*Rattus norvegicus*	RFRP-1	SVTFQELKDWGAKKDIKMSPAPANKVPHSAAN**LPLRFa**	B, E		(36)
		RFRP-3	ANMEAGTMSHFPS**LPQRFa**	B	GnRH1, kisspeptin	(34, 37–39)
Syrian golden hamster	*Mesocricetus auratus*	RFRP-1	SPAPANKVPHSAAN**LPLRFa**	B		(34)
		RFRP-3	TLSRVPS**LPQRFa**	B	GnRH1	(34, 40)
Cow	*Bos taurus*	RFRP-1	SLTFEEVKDWAPKIKMNKPVVNKMPPSAAN**LPLRFa**	B		(41)
		RFRP-3	AMAHLPLRLGKNREDSLSRWVPN**LPQRFa**	B, P		(42)
Sheep	*Ovis aries*	RFRP-1	SLTFEEVKDWGPKIKMNTPAVNKMPPSAAN**LPLRFa**	B		(43, 44)
		RFRP-3	VMAHLPLRLGKNREDSLSRRVPN**LPQRFa**	B, P	GnRH1, NPY, POMC, orexin, MCH	(43–46)
Pig	*Sus scrofa*	LPXRF-1	SLNFEELKDWGPKNVIKMSTPVVNKMPPLAAN**LPLRFa**	B, M, O, E, K, A, U, Pg		(47)
		LPXRF-3	AIASLPLRFGRNTEDSMSRPVPM**LPQRFa**			

*^a^B, brain; P, pituitary; E, eye; T, testis; O, ovary; M, muscle; K, kidney; SP, spleen; Gl, gill; A, adrenal gland; U, uterus; Pg, parotid gland*.

*The identical C-terminal LPXRFamide (X = Leu or Gln) motif sequences are in bold font*.

#### Jawless and jawed fish

In jawless fish species, GnIH orthologs have been identified and characterized in the lamprey (11) and the hagfish (12).

In jawed fish, teleosts GnIH orthologs have been identified and characterized in several species including the goldfish (13), sockeye salmon (48), grass puffer (15), tilapia (16), stickleback, tetraodon, medaka, Takifugu, and the zebrafish (14).

In this review article, all LPXRFa family of peptides (GnIH, RFRP3, and LPXRFa) are designated as GnIH orthologs based on their “GnIH peptide-like” structure. In most fish species, GnIH gene sequence encodes three putative peptide sequences (LPXRFa-1, -2, and -3), while only two putative sequences (LPXRFa-1 and -2) are present in some teleosts such as the stickleback, tetraodon, and takifugu (14). This suggests that the structures of GnIH family of peptides are evolutionarily conserved in vertebrates.

#### Amphibians

In the bullfrog, frog GH-releasing peptide (fGRP) has been identified as the amphibian GnIH orthologous peptide (17). In addition, using the molecular approach, another three fGRP-related peptides (fGRP-RP-1, -RP-2, and -RP-3) have been identified (19). In the European green frog, *Rana* RFamide (R-RFa) with LPXRFa motif has been identified (20). In the newt, four LPXRFa peptides (nLPXRFa-1, -2, -3, and -4) are predicted to be encoded in the newt LPXRFa cDNA. HPLC analysis further confirmed the existence of all four mature LPXRFa peptides in the newt brain (21).

#### Birds

GnIH peptides have been identified in various avian species such as chicken, zebra finches, starlings, and sparrows (10, 24, 28, 29).

#### Mammals

Orthologs of GnIH have also been determined in the mammalian species (43, 49, 50). In mammals, three different RFamide-related peptides (RFRP), including RFRP-1, -2, and -3, were initially identified from the bovine and human brain cDNA, whereas only two RFRPs (RFRP-1 and/or RFRP-3) were discovered in rodents (51, 52). The mammalian GnIH orthologs, RFRP-1 and -3, possess the LPXRFamide (X = Leu or Gln) peptide, which is absent in the RFRP-2 ortholog (53). Therefore, it has been concluded that RFRP-1 and RFRP-3 serve as the functional mammalian GnIH orthologs.

### GnIH receptor

**Figure 2:**
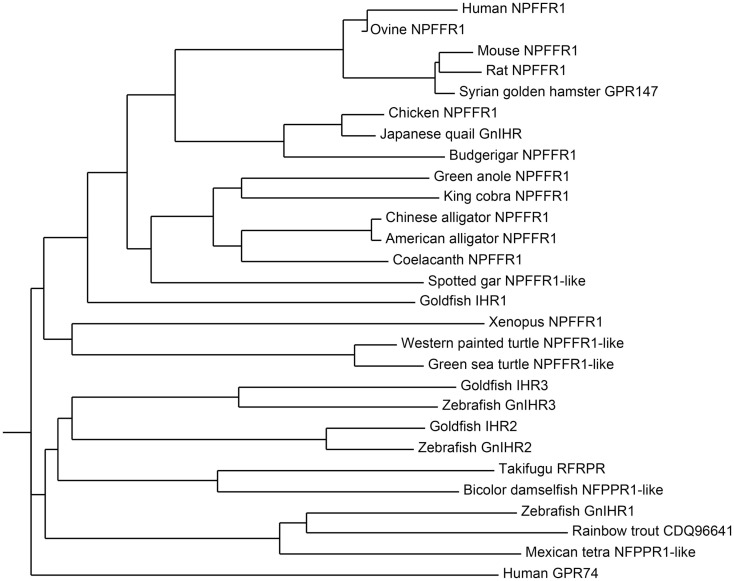
**Phylogenetic tree of GnIH receptor (GPR147) and its homologous sequences in vertebrates**. The phylogenetic tree was constructed by MEGA 3.1 using the neighbor-joining method. GenBank accession numbers for the sequences are listed in Table 2.

The receptor for GnIH family of peptides belongs to the seven transmembrane G protein-coupled receptor (GPCR or GPR) family. Two potential GnIH receptors (GPR147 and GPR74) have been identified in vertebrates and GPR147 has been accepted as a potent receptor for GnIH. The summary of GnIH-homologous peptides and its receptor (GnIHR = GPR147) and its orthologs in various vertebrates and their phylogenetic relationship are demonstrated in Table 2 and Figure 2.

**Table 2:** **List of GnIH receptor (GPR147) and its homologous sequences found or predicted from jawless fish to mammals**.

Animal	Species	Name	GenBank accession number	Distribution^a^	Expression in GnRH or other neurons	Reference
**JAWED FISH**						
Coelacanth	*Latimeria chalumnae*	Neuropeptide FF receptor 1	XP_005991458			Predicted
Spotted gar	*Lepisosteus oculatus*	Neuropeptide FF receptor 1 like	XP_006630407			Predicted
Goldfish	*Carassius auratus*	G-protein couple receptor IHR1/GnIHR1	AFY63167	B, P, T, O		(54, 55)
		G-protein couple receptor IHR2/GnIHR2	AFY63168	B, P, T, O		(54, 55)
		G-protein couple receptor IHR3/GnIHR3	AFY63169	B, P		(54)
			AER11372	
Zebrafish	*Danio rerio*	GnIHR1 (neuropeptide FF receptor 1 like 1)	ADB43133	B, P, T, M, K, SP, H, Gl, E		(14)
			NP_001165167	
		GnIHR2 (neuropeptide FF receptor 1 like 2)	ADB43134	B, T, K, SP, H, L, Gl, E		(14)
			NP_001165168	
		GnIHR3 (neuropeptide FF receptor 1)	ADB43135	B, T, O, M, K, SP, IN, H, Gl, E		(14)
			NP_001082858	
Takifugu	*Takifugu rubripes*	RFamide-related peptide receptor	BAF34887	B, P, E, K		(25)
Mexican tetra	*Astyanax mexicanus*	Neuropeptide FF receptor 1 like	XP_007255089			Predicted
Rainbow trout	*Oncorhynchus mykiss*	Unnamed protein product	CDQ96641			(56)
Bicolor damselfish	*Stegastes partitus*	Neuropeptide FF receptor 1 like	XP_008295983			Predicted
**AMPHIBIAN**						
Xenopus	*Xenopus laevis*	Neuropeptide FF receptor 1	NP_001084551			(57)
**REPTILE**						
Green anole	*Anolis carolinensis*	Neuropeptide FF receptor 1	XP_008104865			Predicted
King cobra	*Ophiophagus hannah*	Neuropeptide FF receptor 1	ETE63534			(58)
Chinese alligator	*Alligator sinensis*	Neuropeptide FF receptor 1	XP_006027961			Predicted
American alligator	*Alligator mississippiensis*	Neuropeptide FF receptor 1	XP_006265135			Predicted
Western painted turtle	*Chrysemys picta bellii*	Neuropeptide FF receptor 1 like	XP_005286579			Predicted
Green sea turtle	*Chelonia mydas*	Neuropeptide FF receptor 1 like	XP_007053537			Predicted
**BIRD**						
Japanese quail	*Coturnix japonica*	GnIH receptor	BAD86818	B, T, O		(23, 59)
European starling	*Sturnus vulgaris*	GnIH receptor	EF212891	B, P, T, O	GnRH1, GnRH2	(23, 28)
Budgerigar	*Melopsittacus undulatus*	Neuropeptide FF receptor 1	XP_005154065			Predicted
Chicken	*Gallus gallus*	Neuropeptide FF receptor 1	NP_989693	B, P, T, O		(25, 60, 61)
			BAE17050	
**MAMMAL**						
Human being	*Homo sapiens*	Neuropeptide FF receptor 1	NP_071429	B, P		(30, 36)
Mouse	*Mus musculus*	Neuropeptide FF receptor 1	NP_001170982		GnRH, kisspeptin	(35, 62, 63)
Rat	*Rattus norvegicus*	Neuropeptide FF receptor 1	NP_071627	B, E	GnRH, kisspeptin, dopamine	(36, 39)
Syrian golden hamster	*Mesocricetus auratus*	GPR147	ACY39880	B, P, T		(64, 65)
Sheep	*Ovis aries*	Neuropeptide FF receptor 1	ABW08098	B		(44)
Pig	*Sus scrofa*	Neuropeptide FF receptor 1	HQ681286	B, P, O, K, E, U, A, IN, S		(47)

*^a^B, brain; P, pituitary; E, eye; T, testis; O, ovary; M, muscle; K, kidney; SP, spleen; Gl, gills; H, heart; L, liver; IN, intestine; A, adrenal gland; U, uterus*.

#### Jawless and jawed fish

In jawless fish, there is no report on identification of GnIH receptor to date. In jawed fishes, GnIH receptors have been identified in several species; where GPR147 has been identified in the grass puffer (15), goldfish (66), zebrafish (14), and tilapia (16), and GPR74 has been identified in several teleosts species (14, 16). In most teleosts, only one GnIH receptor gene has been identified, while in the zebrafish, three different GnIH receptor gene types (*gnihr1*, *gnihr2*, and *gnihr3*) have been isolated (14). However, the binding affinities of teleost GPR147 and GPR74 to GnIH peptides have not been characterized. Our recent study has shown that tilapia GPR147 (tiLPXRFa-R) has strong affinity to tilapia LPXRFa-2 peptides through both cAMP/PKA and Ca^+2^/PKC pathways (16).

#### Birds

In the avian species, two receptors (GPR74 and GPR147) have been identified and further characterization has revealed GPR147 as the potent receptor for the avian GnIH based on their binding affinity to GnIH and RFRP-3 peptides (25, 59).

#### Mammals

In mammals, two receptors (GPR74 and GPR147) have been identified (36, 44, 67, 68). GPR147 couples to G_αi_ protein, which is involved in inhibiting the production of cAMP (36). Therefore, GPR147 is generally accepted as the candidate receptor for GnIH and RFRP-3 in birds and mammals because of its stronger inhibitory effect on G_αi_ mRNA expression in COS-7 cells, as compared to that of GPR74 (25, 52, 69). However, other studies have shown that GPR147 receptor also tends to bind to Gα_i3_ and Gα_s_ proteins, while GPR74 binds to Gα_i2_, Gα_i3_, Gα_o_, and Gα_s_ proteins (70).

## Distribution of GnIH and GnIHR

### Distribution of GnIH neurons in the brain

**Figure 3:**
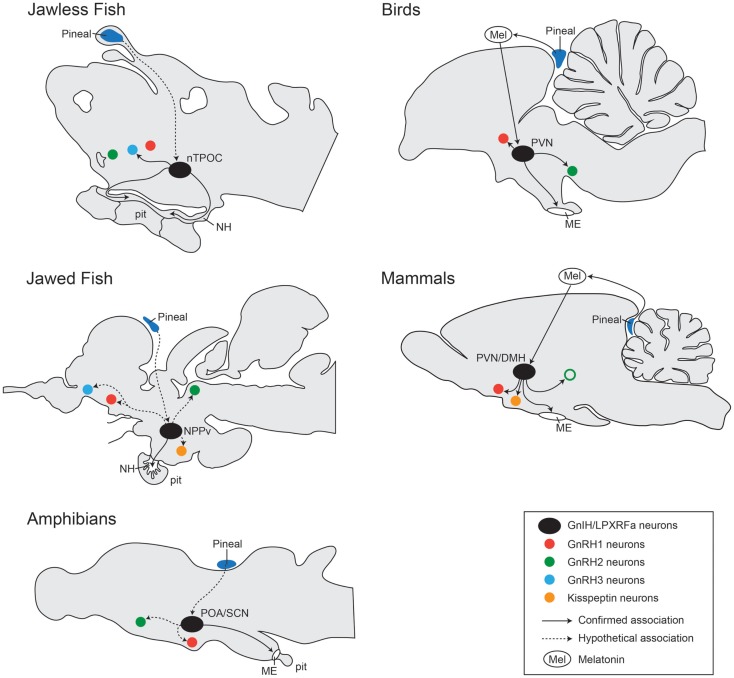
**Comparison of localization of GnIH cells and their associations with other neural systems in the brain from jawless fish, jawed fish, amphibians, birds, and mammals**. In jawless fish (sea lamprey), LPXRFa-cells are localized in the bed nucleus of the tract of the postoptic commissure (nTPOC) in the hypothalamus (11). In jawed fish (goldfish, salmon, and carp), LPXRFa-immunoreactive cells are seen in the nucleus posterioris periventricularis (NPPv) (13, 48, 72). In amphibians (bullfrog and newt), LPXRFa-neurons are seen in the anterior preoptic area (POA) and/or the suprachiasmatic nucleus (SCN) (17, 18, 20, 21). In birds, the GnIH neurons are present in the PVN. In mammals and birds, GnIH neurons project to the median eminence (ME). In mammals, GnIH neurons are localized in the dorsomedial nucleus of the hypothalamus (DMH) and in the paraventricular nucleus (PVN). In jawless fish (sea lamprey), LPXRFa-immunoreactive fibers are seen in the neurohypophysis (NE), suggesting action of GnIH on the pituitary (pit) cells. In jawed fish, LPXRFa-immunoreactive fibers are present in the pituitary. In amphibians, birds, and mammals, LPXRFa/GnIH fibers are terminated in the ME. In birds and mammals, GnIH cells (*black circle*) associated with several other neurons such as GnRH neuron types: GnRH1 (*red circle*), GnRH2 (*green circle*), and GnRH3 (*blue circle*) neurons. The open circle with green indicates the presence of GnRH2 neurons only in certain mammalian species such as primates but not in rodents (73). In some mammals, GnIH fibers are also closely associated with kisspeptin neurons (*yellow circle*). GnIH neural functions are regulated by melatonin (Mel) derived from the pineal gland (dark blue) or eyes. In jawed and jawless fish, the effect of melatonin on GnIH neurons is still unknown, but there might be direct projection from the pineal gland to GnIH neurons in the hypothalamus (74–76). Confirmed association is indicated by the line, and unconfirmed hypothetical association is indicated by the dotted line.

Compared to mammals and birds, in other non-mammalian vertebrate species, studies describing the distribution of GnIH expression are very few due to limited GnIH gene sequences and the lack of specific antibodies to non-mammalian GnIH orthologous peptides. The distribution pattern of GnIH neurons in the brain of various vertebrate species are illustrated in Figure 3 (71).

#### Jawless and jawed fish

In the brain of sea lamprey, the expression of lamprey LPXRFa mRNA as well as lamprey LPXRFa-immunoreactive cells has been detected in the bed nucleus of the tract of the postoptic commissure (nTPOC) in the hypothalamus (11). Lamprey LPXRFa-immunoreactive fibers are widely seen in the brain and a few fibers are seen in the neurohypophysis (11).

In jawed fish species, such as the goldfish, *in situ* hybridization study has shown the expression of GnIH mRNA in the nucleus posterioris periventricularis (NPPv) in the hypothalamus (13). Using antibodies to avian GnIH and fGRP, the distribution of GnIH orthologs-like immunoreactivity has been examined in the brain of several teleosts including the goldfish (13), sockeye salmon (48), and the Indian major carp (72). In the sockeye salmon and the Indian major carp, the distribution pattern of fGRP/GnIH-immunoreactive cells is similar to GnIH mRNA expression in the NPPv of the goldfish (13, 48, 72), suggesting that the presence of GnIH neurons in the NPPv is a common pattern in teleosts.

The presence of fGPR/GnIH-immunoreactive fibers have been reported in several brain regions including in the olfactory bulb, telencephalon, optic tectum, mesencephalon, diencephalon, and the spinal cord (13, 48, 72). In the goldfish and sockeye salmon, the presence of fGPR-immunoreactive fibers has also been noted in the pituitary (13, 48). In the pituitary of the Indian major carp, GnIH-immunoreactive cells and fibers have been detected in the proximal pars distalis region only during the early developmental stage, but not in adults (72). However, in the Indian major carp, GnIH-immunoreactive cells are also seen in several mesencephalic regions, such as the nucleus of medial longitudinal fascicle and the occulomotor nucleus (72), which needs further verification by *in situ* hybridization with specific GnIH gene sequence in the Indian major carp. Similarly, in the goldfish, fGRP-immunoreactive cells have been reported in the terminal nerve of the olfactory bulb, where no GnIH mRNA is expressed (13), which indicates the fGRP antibody has cross reactivity to other unknown RFamide peptides. Therefore, to identify the targets of GnIH neurons in the brain and in the pituitary more precisely, a specific antibody to fish GnIH orthologs peptide needs to be generated.

#### Amphibians

In the brain of the European green frog, R-RFa-containing neurons are localized in the hypothalamus, which includes the anterior preoptic area (POA), the suprachiasmatic nucleus (SCN), and the dorsal and ventral hypothalamic nuclei (20). R-RFa-containing fibers are widely distributed throughout the brain from the olfactory bulb to the brainstem, and are particularly abundant in the external layer of the ME (20). In the bullfrog, fGRP neurons are mainly seen in the telencephalon and the diencephalon including the medial septum, nucleus of the diagonal band of Broca, anterior POA and the SCN (17, 18). fGRP-immunoreactive fibers are widely distributed throughout the brain including mesencephalic and rhombencephalic regions, and are terminate in the ME (17). In the newt brain, nLPXRFa mRNA and the peptide (with anti-fGRP serum) are expressed only in the SCN in the hypothalamus (21). Similar to frogs, fGRP-immunoreactive fibers are seen in the mesencephalic and rhombencephalic regions and terminate in the ME (21).

#### Reptiles

In the Japanese grass lizard, GnIH-immunoreactive neurons are seen in the nucleus accumbens, paraventricular nucleus (PVN), and upper medulla, and GnIH fibers are distributed in the third ventricle, the paraventricular organ, and the ME (77).

#### Birds

In the avian species, majority of the hypothalamic GnIH neuronal cell bodies are present in the PVN, with the main projections extending to the ME (10, 26, 78, 79). However, in the ME of Rufous-winged sparrows, there are no GnIH fibers (80), although expression of GnIH receptors has been shown in the pituitary (69). Additionally, the diencephalic and mesencephalic regions of the avian brain have extensive distribution of GnIH fibers.

#### Mammals

In rodents, GnIH neurons are concentrated within the dorsomedial nucleus of the hypothalamus (DMH), where abundant fibers project to the hypothalamic and limbic structures (34). In the ovine species, GnIH neurons are widespread in the brain, where they are present throughout the DMH, PVN, and the mediobasal hypothalamus (43). Recently, using transgenic rats carrying an enhanced green fluorescent protein (EGFP) tagged to the GnIH promoter, another population of smaller EGFP-positive neurons were seen in the ventromedial hypothalamus (VMH), which was not detected previously by GnIH immunohistochemistry (81). The mammalian GnIH fiber terminals project to the external layer of the ME (30, 31, 43, 64), suggesting the action of GnIH on the pituitary via the blood vasculature, which is supported by the measurement of GnRH peptide concentration in hypophyseal portal blood in ewes (82). However, GnIH-immunoreactive fibers are absent in the ME of hamsters (34, 40) and Wistar rats (83).

### Distribution of GnIH receptors in the brain and pituitary

In most vertebrates, GnIH receptors (GPR147) are mainly expressed in the pituitary and in several brain regions including the hypothalamus and the spinal cord (14, 25, 30, 59, 84), most of which have been examined mainly by RT-PCR or Southern-blot analysis. However, to date, detail neuroanatomical information of GnIH receptor localization in the vertebrate brain is very limited (28).

#### Jawless and jawed fish

There is no report demonstrating the distribution of GnIH receptor in jawless species. However, in jawed fish species, the zebrafish, the expression of three GnIH receptors have been detected in the brain by RT-PCR (14). In the zebrafish, two GnIH receptors genes (*gnihr1* and *gnihr3*) are expressed in the pituitary (14). In the grass puffer and the tilapia, both GnIH and GnIH receptor genes are expressed in the brain and pituitary (15, 16). Furthermore, our recent study in the tilapia has shown the co-expression of GnIH receptor gene (*lpxrf-r*) in LH and FSH cells by double *in situ* hybridization (16).

#### Birds and mammals

In the quail, RT-PCR has shown GnIH receptor mRNA expression in the cerebrum, diencephalon, mesencephalon, and the spinal cord (59). In human beings, the expression of GnIH receptor gene has been shown in the hypothalamus and in the pituitary by RT-PCR (30). In the human pituitary, gene expression of GnIH receptors in LH cells has been shown by *in situ* hybridization (30).

### Distribution of GnIH and GnIH receptors in the gonads

In several vertebrate species, the expression of GnIH and GnIH receptors has been reported in some peripheral tissues including the gonadal tissues (69) (Tables 1 and 2), indicating the role of GnIH in ovarian or testicular maturations (65, 85). Expression of GnIH and/or GnRH receptor has been shown in the gonadal tissues by RT-PCR, *in situ* hybridization, and immunohistochemistry (32, 86).

#### Jawless and jawed fish

In the sea lamprey, LPXRFa mRNA is expressed in the testis and ovary (11).

In the zebrafish, GnIH and three GnIH receptor genes (*gnihr1*, *gnihr2*, and *gnihr3*) are expressed in the testis, and GnIH and GnIH receptor gene (*gnihr3*) are expressed in the ovary (14). Similarly, in the goldfish, two out of three GnIH receptor types (*gnrh1* and *gnrh2*) are expressed in the testis and ovary (55). In the tilapia, LPXRFa and LPXRFa-R (GPR147) mRNAs are expressed in the gonads (16). However, in the grass puffer, there is no expression of LPXRFa and LPXRFa-R mRNAs in the gonads (15). *In situ* hybridization study in the goldfish has shown expression of *gnrh1* and *gnrh2* genes in the oocytes only before the cortical alveolus stage, but not at the vitellogenic stage (55). In the testis of goldfish, expression of two GnIH receptor gene types have been reported in the interstitial tissue (55). *In vitro* treatment of goldfish gonadal cell culture with GnIH peptides (gfLPXRFa-2 and gfLPXRFa-3) has no effect on the mRNA expression of genes involved in steroidogenesis in ovarian cells, while in testicular cell culture, GnIH peptides significantly upregulate the expression of genes involved in testosterone biosynthesis, but suppress the CYP9 gene, which is responsible for aromatization of testosterone (55).

#### Amphibians and reptiles

There is no report demonstrating the presence of either GnIH or GnIH receptors in gonadal tissues of amphibian species.

In reptiles, the garden lizard, *Calotes versicolor*, has GnIH-immunoreactivity in the granulosa cells of previtellogenic follicles and stroma cells, which is relatively higher during inactive phase, but lower during the active preovulatory phase suggesting inverse correlation with circulating estradiol level (87).

#### Birds

In birds, GnIH and GnIH receptor gene expression has been shown in the testis and ovary by RT-PCR (23, 60, 88). Furthermore, *in situ* hybridization and immunohistochemical approaches have revealed the presence of GnIH mRNA and peptides in the ovarian thecal and granulosa cells, testicular interstitial and germ cells, and pseudostratified columnar epithelial cells in the epididymis (23, 88). GnIH receptor is also localized in the ovarian thecal and granulosa cell layers, and testicular interstitial, germ cells, and spermatocytes (23, 60, 88). In the European starlings, melatonin upregulates the expression of GnIH mRNA in the gonads. Furthermore, GnIH and melatonin significantly decrease testosterone secretion from LH/FSH-stimulated testes (89), suggesting that GnIH is involved in the seasonal regulation of testicular maturation.

#### Mammals

In the mammalian species, the expression of GnIH and GnIH receptors and the role of GnIH in gonadal maturation have been well demonstrated (32, 85). In the Syrian hamster, the presence of GnIH and GnIH receptor has been shown in spermatocytes and in spermatids, but not in the Leydig cells of the testis (65). In the rhesus macaque, GnIH and GnIH receptors are expressed in the Leydig cells, spermatogonia, and spermatocytes, and in the ovarian preantral follicles and granulosa cells (88). In the ovary of mice, GnIH is expressed in the granulosa cells, antral follicles, and the luteal cells (90). Similarly, in the pig, GnIH and GnIH receptor immunoreactivity has been shown in the luteal cells and in the granulosa and theca cells of the antral follicles during proestrus and estrus (47). In human beings, the expression of GnIH and GnIH receptor has been shown in the granulosa cell layer of large preovulatory follicles and the corpus luteum as well as in the primary cultures of human granulosa-lutein cells (91). A very recent study in mice has reported that GnIH (RFRP-3) treatment reduces germ cell proliferation and survival but increases apoptosis with a reduction of testosterone synthesis in the testis in a dose-dependent manner (92). Similarly, mice treated *in vivo* with GnIH for 8 days show dose-dependent changes in ovarian follicular morphology, reduction in the number of healthy antral follicles, an increase in the number of atretic follicles with low dose of GnIH (100 ng/day), and appearance of abnormal follicles at high doses (2 μg/day) (93). *In vitro* treatment of mice ovary with GnIH suppresses the production of ovarian progesterone synthesis and reduces steroidogenic enzymes such as 3β-hydroxysteroid dehydrogenase (93).

### Association of GnIH system with other neural systems

Based on the morphological distribution of GnIH and GnIH receptors in the brain and pituitary, their potential role as well as their mechanism of action have been well demonstrated in the avian and the mammalian species. In birds and mammals, GnIH fibers are seen in close proximity to the GnRH neurons in the POA (22, 28, 30, 45, 78–81) (Figure 3). Furthermore, the expression of GnIH receptor has been shown in GnRH1 neurons (28, 40, 94–96). In monkeys and birds, GnIH neurons send projections to midbrain GnRH2 neurons that express GPR147 (28, 30, 78). However, in ray-fin fishes, neural associations between GnIH with other hypothalamic neurons are very limited due to the lack of specific antibody.

#### Jawless and jawed fish

In the sea lamprey, lamprey GnIH (LPXRFa-2) immunoreactive fibers have been observed in close apposition to GnRH-III neurons (11).

A recent study in the dwarf gourami demonstrated that medaka GnIH (RFRP2 = LPXRFa-2) inhibits the pacemaker activity of GnRH3 neurons in the terminal nerve (97), suggesting the functional association of GnIH fibers with non-hypothalamic GnRH3 neurons. This suggests the action of GnIH on GnRH neurons could be evolutionarily conserved in vertebrates, which remains to be further confirmed in other fish species with fish-specific GnIH antibodies.

#### Birds

Interactions of GnIH with GnRH1 (c-GnRH-I) neurons are seen in several avian species including the Japanese quail, European starling, song sparrow, house sparrow, and the zebra finch (22, 28, 29, 34, 78). In Gambel’s white-crowned sparrow and European starling, GnIH fibers are also closely associated with GnRH2 (c-GnRH-II) neurons (27, 28). Furthermore, expression of GnIH receptor mRNA has been identified in GnRH1 and GnRH2 neurons in the brain of the European starling (28).

#### Mammals

In the rhesus macaque, GnIH fibers are observed in close proximity to GnRH1 and GnRH2 neurons (31). A morphological study in the sheep using a retrograde tracer has shown fiber projection of GnIH neurons to several other hypothalamic neuropeptides-containing neurons, such as to neuropeptide Y, pro-opiomelanocortin (POMC), orexin, melanin-concentrating hormone, corticotrophin-releasing hormone, and oxytocin neurons (46). Similarly, GnIH fibers are seen in close association with POMC neurons in mice (98). In rats, GnIH fibers are closely associated with kisspeptin neurons in the rostral periventricular region of the third ventricle region (39), and in the arcuate nucleus of mice (35), which is supported by the expression of GPR147 mRNA in kisspeptin neurons (35, 95). On the other hand, very few GnIH cells (3–7%) receive kisspeptin fibers in mice (35). Interestingly, in mice, GnIH neurons also co-express neurokinin B (*Tac2*) and its receptor (*Tacr3*) mRNAs (35).

In addition to neuropeptides, GnIH neurons are also associated with neurotransmitters. In the rhesus macaque, GnIH fibers are closely associated with dopamine and β-endorphin neurons (31). In mice, morphological and electrophysiological studies have revealed functional interactions between GnIH with glutamatergic neurons but not with cholinergic or GABAergic neurons (99). In rats, GPR147 is expressed in dopamine neurons (36). In addition, a recent report in rats has shown no co-expression of GnIH neurons with GABA (39). In rats, GnIH neural population in the DMH express 11 types of serotonin receptors (63). Similar observation has been reported in the Japanese grass lizard (77). These results indicate multiple functions of the GnIH system, in addition to its inhibitory action on reproduction.

## Function of GnIH-GnIHR Signaling in Vertebrate Reproduction

### Role of GnIH in gonadotropin synthesis and release

As the name of the peptide indicates, GnIH peptides act as inhibitory factor in the control of reproduction mainly in birds and mammals (10, 34). Similar findings have been reported in various vertebrate species (52) (Table 3). On the contrary, in ray-fin fishes, the role of GnIH peptides in the control of gonadotropin release has been debatable.

**Table 3:** **Functions of GnIH and its homologous peptides from jawless fish to mammals**.

Animal	Species	GnIH types	Functions	Reference
**JAWLESS FISH**				
Sea lamprey	*Petromyzon marinus*	LPXRFa-2	Stimulation of GnRH-III synthesis and GTHβ mRNA expression	(11)
**JAWED FISH**				
Goldfish	*Carassius auratus*	gfLPXRFa-1	Stimulation of GTH and GH release	(48)
		gfLPXRFa-2	Stimulation of GTH and GH release	(48)
			Inhibition of LH and FSH synthesis	(54)
		gfLPXRFa-3	Stimulation of GTH and GH release	(48)
			Inhibition of LH synthesis	(54)
			Inhibition of GnRH-elicited FSH synthesis	(54)
			Stimulation of GTH synthesis and release in prespawning fish	(66, 100)
			Inhibition of GTH synthesis in early to later stages of gonadal recrudescence	(100)
			Inhibition of GnRH-elicited GTH synthesis in early and mid gonadal recrudescence	(66)
Zebrafish	*Danio rerio*	gfLPXRFa-3	Inhibition of GTH release	(14)
Grass puffer	*Takifugu niphobles*	gfLPXRFa-1	Stimulation of GTH synthesis	(15)
Nile tilapia	*Oreochromis niloticus*	LPXRFa-2	Stimulation of LH and FSH release (*in vivo* and *in vitro*)	(16)
**AMPHIBIAN**				
Bullfrog	*Rana catesbeiana*	fGRP	Stimulation of GH release	(17)
		fGRP-RP-2	Stimulation of GH/PRL release	(19)
**BIRD**				
Japanese quail	*Coturnix japonica*	GnIH	Inhibition of GTH synthesis and release	(10, 101)
Chicken	*Gallus gallus*	GnIH	Inhibition of GTH synthesis and release	(102)
			Inhibition of LH release in immature but not mature chickens	(60)
			Stimulation of feeding behavior	(103)
		GnIH-RP-1	Stimulation of feeding behavior	(103)
		GnIH-RP-2	Stimulation of feeding behavior	(103)
Gambel’s white-crowned sparrow	*Zonotrichia leucophrys* gambelii	GnIH	Inhibition of GnRH-elicited GTH release	(26, 27)
			Inhibition of reproductive behavior	
Song sparrows	*Melospiza melodia*	GnIH	Inhibiting GnRH-induced LH release	(26)
Rufous-winged sparrow	*Aimophila carpalis*	GnIH	No effect on LH release and GnRH-elicited LH secretion	(104)
**MAMMALS**				
Human being	*Homo sapiens*	RFRP-1	Stimulation of PRL release	(36)
Mouse	*Mus musculus*	RFRP-3	Suppressive action on the excitability of GnRH neurons	(105)
Rat	*Rattus norvegicus*	RFRP-1	Stimulation of ACTH and oxytocin release	(106)
		RFRP-3	Stimulation of ACTH and oxytocin release	(106)
			Stimulation of GH secretion	(38)
			Inhibition of GTH release	(38, 49)
			Inhibition of GnRH-elicited GTH release	(49)
			Inhibition of reproductive behavior	(38)
			Stimulation of feeding behavior	(38, 49)
			No effect on basal LH secretion, but inhibition of GnRH-elicited LH release	(83)
Syrian golden hamster	*Mesocricetus auratus*	RFRP-1	Inhibition of GTH release	(34)
		RFRP-3	Inhibition of GTH release	(34, 40, 64)
Bovine	*Bos taurus*	RFRP-3	Inhibition of GnRH-elicited GTH release	(50)
Ovine	*Ovis aries*	RFRP-3	Inhibition of GnRH-elicited GTH synthesis and release	(43, 107)
			Reduction of the amplitude of LH pulses	

#### Jawless and jawed fish

In female lampreys, treatment of lamprey GnIH (LPXRFa-2) stimulates the expression of lamprey GnRH-III protein in the hypothalamus and GTHβ mRNA expression in the pituitary (11).

The first physiological study demonstrating the role of teleost GnIH peptides (goldfish LPXRFa-1, -2, and -3 peptides) was reported in the sockeye salmon, in which goldfish LPXRFa peptides increase the release of LH, FSH, and growth hormone (GH) from cultured pituitary cells (48). Similarly, an *in vivo* study in the goldfish has shown that GnIH significantly increases pituitary levels of mRNAs for LHβ and FSHβ in a reproductive stage-dependent manner (100). Goldfish GnIH (gfLPXRFa-1) peptide treatment to the grass puffer significantly stimulates FSHβ and LHβ gene expression (15). Our recent study in the female tilapia has shown that tilapia LPXRFa-2 peptides positively increase LH and FSH release *in vitro* and *in vivo* (16).

In contrast, intraperitoneal administration of zebrafish GnIH (LPXRFa-3) to goldfish decreases the plasma LH levels (14). Similarly, inhibitory effects of GnIH on circulating serum LH levels have been demonstrated during the early to later stages of recrudescence in the goldfish (66, 100). These differences in gonadotropin responses to GnIH seen in different and in the same fish species (summarized in Table 3) can be explained by a recent physiological study conducted in the goldfish (54). Intraperitoneal injections of goldfish GnIH-II peptide and GnIH-III peptide significantly decreases FSHβ mRNA levels, whereas *in vitro* application of GnIH has no effect on gonadotropin synthesis. However, an inhibition of GnRH-stimulated LHβ and FSHβ synthesis has been observed when GnIH-III was applied to primary pituitary cell cultures (54). Collectively, these reports in ray-fin fish species suggest that the inhibitory action of GnIH on gonadotropin synthesis/release is closely associated with the reproductive stages in fish, which can be modulated by GnRH-dependent mechanism of action as in birds and mammals (26, 95).

#### Birds and mammals

In birds and mammals, GnIH reduces gonadotropin release from the anterior pituitary (10, 34), which has been extensively reviewed previously. RFRP-3 inhibits the synthesis and/or release of gonadotropins across various mammalian species, and recently, it has also been found that RFRP-1 is capable of inhibiting the release of gonadotropins in hamsters (40). Indeed, in sheep, GnIH (GnIH-3) peptide levels in the portal blood are around 2–3 pg/ml during the breeding season but increase to 4–8 pg/ml during the non-breeding season (82). In rats, the central administration of GnIH (RFRP3–8) peptides has shown to suppress the circulating LH levels at the dose of 1 nmol/injection *in vivo*, and GnIH suppresses gonadotropin secretion from pituitary culture at the concentration of 10^-8^ M *in vitro* (108). However, in rufous-winged sparrows (*Aimophila carpalis*), there is no effect of peripheral injections of GnIH on basal plasma LH levels and on GnRH-elicited LH secretion (104). This could be due to the shorter half-life of GnIH peptides *in vivo* compared with *in vitro*. In ewes, the half-life of peripherally injected GnIH in portal blood is 6.03 ± 0.30 min *in vivo* (82). While under *in vitro* condition, the half-life of GnIH (RFRP3–8) peptides is 14.3 min in rat serum (108).

### Role of GnIH in socio-sexual behaviors

Gonadotropin-inhibitory hormone is also involved in the regulation of reproductive and social behaviors (Table 3) (109).

#### Jawless and jawed fish

The role of GnIH orthologs in socio-sexual behaviors has not been demonstrated in jawless and jawed fish species. Nevertheless, a recent study has suggested GnIH as a regulator of neuroestrogen synthesis (110) and the potential involvement of neuroestrogen in socio-sexual behaviors has been demonstrated in several jawed fish species. In a sex-changing fish (*Lythrypnus dalli*), socially induced decrease in brain aromatase levels correspond with increased aggression (111). Male Endler guppy (*Poecilia reticulata*) treated with the aromatase inhibitor show reduce of courtship activities (112). In the African cichlid fish (*Astatotilapia burtoni*), males treated with aromatase inhibitor show decrease aggressive, but not reproductive behaviors (113).

#### Birds

Female white-crowned sparrows injected with GnIH show inhibition of copulation-solicitation with the reduction of circulating LH levels (27). In the European starlings, there is close association between social and breeding status and GnIH levels in the brain (114). Indeed, bird pairs (male and female) with nest (winner) have significantly different numbers of GnIH peptide-producing cells than those without nest (losers), suggesting that GnIH may play a key role in the switch from mating and aggressive behaviors to those of parental care (114). Similarly, in the male quail, the role of GnIH in aggressive and sexual behaviors has been demonstrated (115), which has been suggested to be regulated by increasing neuroestrogen synthesis (110).

#### Mammals

In rats, GnIH injections suppress male sex behaviors (38). On the contrary, in a study in non-human primates, ewes, and rats, there is no effect of GnIH on sexual behavior (116), which could be due to different injection conditions (109). In the female Syrian hamsters, GnIH treatment inhibits sexual motivation and precopulatory behavior, but has no effect on copulatory behavior (117). GnIH is critical for the regulation of socio-sexual arousal, motivation, and performance in vertebrates (110). Therefore, changes in socio-sexual behaviors that are influenced by neuroestrogen levels can be modulated by GnIH in fish as in birds.

### Regulators of GnIH system

In addition to the role of GnIH, its regulatory mechanism has also been well examined (52, 85, 118). For example, GnIH neurons express steroid receptors (ERα and AR), which are responsible for steroid response in GnIH neurons (34, 119). There are numerous factors that suppress reproduction and these have been demonstrated as regulators of the GnIH system. GnIH system is known to be regulated by environmental cues particularly seasonal- and diurnal-rhythmicity (120–122). Furthermore, seasonal or photoperiod-dependent alterations of GnIH neurons indicate the modulatory role of melatonin in GnIH expression and synthesis (123).

#### Seasonal regulation

##### Jawless and jawed fish

Seasonal effect on GnIH orthologs has not been demonstrated in jawless fish species. In the goldfish, the effect of GnIH injections on the reduction of circulating LH levels is closely associated with seasonal dependent gonadal maturation stages (100). Interestingly, in the grass puffer, GnIH and GnIH receptor gene expression patterns are synchronized with diurnal and circadian rhythmicity, which indicates the involvement of GnIH system in the regulation of lunar-synchronized spawning (15). Furthermore, the potential neuronal mechanism of seasonal-dependent change in GnIH system has been demonstrated (15). However, there is no direct evidence that demonstrates melatonin action on GnIH in fish, although the role of melatonin in the regulation of fish reproduction has been well recognized (124, 125). Nevertheless, in some teleosts species, there is direct projection from the pineal organ to the NPPv in the hypothalamus (74, 75), where GnIH neurons exist in teleost species. These results indicate that the GnIH system plays an important role to transmit photoperiodic cues via melatonin signaling in vertebrate reproduction.

##### Amphibians

In newts, peripheral treatment (intraperitoneal injection) of melatonin (at 1 h post-injection) or treatment in water containing melatonin (for 2 weeks) induces LPXRFa gene expression in the brain (21). Similarly, in bullfrogs, fGRP neurons in the SCN express Mel_1b_, a melatonin receptor subtype (18). Furthermore, the expression of fGRP precursor mRNA is photoperiodically controlled, which increases under short-day photoperiods, when the nocturnal duration of melatonin secretion increases (18), suggesting stimulatory action of melatonin on fGRP secretion.

##### Birds

In the song sparrows, GnIH peptide levels are highest at the end of the breeding season (78). Similarly, in the Rufous-winged sparrow (*A. carpalis*), male birds during the breeding season have fewer, less densely labeled GnIH cell bodies than birds before the breeding season (80). While in the Australian zebra finches (*Taeniopygia guttata*), GnIH cell number and size, as well as GnIH mRNA levels are similar in the breeding and the non-breeding conditions (126). In the Japanese quail, GnIH mRNA levels decrease significantly in the pinealectomized birds (127). Furthermore, melatonin administration causes a dose-dependent increase in the expression of GnIH precursor mRNA as well as the production and release of mature peptide, which is modulated via Mel_1c_ receptor subtype (127, 128). Interestingly, in the song birds, the pineal gland conveys photoperiodic information to the vocal control system to regulate song behavior (129). Furthermore, a recent study in female great tits (*Parus major*) has shown that melatonin treatment delays clutch initiation (130). Interestingly, one of the song-control nucleus in the telencephalic area, called area X is sensitive to melatonin (131) and GnIH neurons may have association with the area X (79). Therefore, it would be interesting to look into the possible association between GnIH system and song behavior.

##### Mammals

Similar to other vertebrates, mammalian GnIH is also influenced by seasonal change. In the sheep, lower expression of RFRP levels in the brain is concurrent with the breeding season (45). In Syrian and Siberian hamsters, RFRP mRNA and the number of RFRP-immunoreactive cell bodies decrease under short-day photoperiod (132). Furthermore, in the Syrian hamsters treated with melatonin (60 days), RFRP mRNA levels significantly decrease in the brain (132).

#### Stress regulation

GnIH has also been demonstrated as a modulator linking stress and reproduction in several vertebrate species. In addition, in birds and mammals, GnIH neurons are sensitive to stress hormones such as glucocorticoid or corticotropin-releasing hormone (CRH) (96, 133, 134).

##### Jawless and jawed fish

There is no report demonstrating the involvement of GnIH in stress response in jawless and jawed fish. However, our promoter prediction search with the ALGGEN PROMO with TRANSFAC database v. 8.3 (135, 136) reveals the presence of a putative glucocorticoids response elements (GRE) at −983 bp upstream of the zebrafish GnIH gene promoter sequence. In addition, there are several putative GRE sites within −2,000 bp upstream of zebrafish GnIHR genes (GnIHR1: at −1,755 and −1,976 bp; GnIHR2: at −30, −260, −265, −344, −1,642, and −1,942 bp; GnRHR3: at −909 and −1,294 bp). These results indicate that the role of GnIH signaling could be evolutionarily conserved in the vertebrates.

##### Birds

In the house sparrows (*Passer domesticus*), there is a significant increase in GnIH positive neurons in stressed birds (137). In the European starlings, plasma corticosterone concentration is positively correlated with GnIH mRNA abundance at the middle of the breeding season (114). In the Japanese quail, corticosterone treatment increases GnIH mRNA expression in the diencephalon (134). Furthermore, glucocorticoid receptor (GR) is expressed in quail GnIH neurons (134).

##### Mammals

In male rats, acute and chronic immobilization stress leads to an upregulation of GnIH gene expression (133). Furthermore, corticosterone treatment increased GnIH mRNA expression in a GnIH-expressing cell line, rHypoE-23, derived from the rat hypothalamus (138), which can be blocked by GR antagonist (134, 139). In male rats, 53% of GnIH neurons co-express GR, and 11.8% of GnIH neurons co-express CRH receptor1 (133). Furthermore, one functional GRE has recently been identified in the promoter region of rat GnIH gene (134), suggesting that corticosterone directly induces GnIH transcription via GR.

## Summary

**Figure 4:**
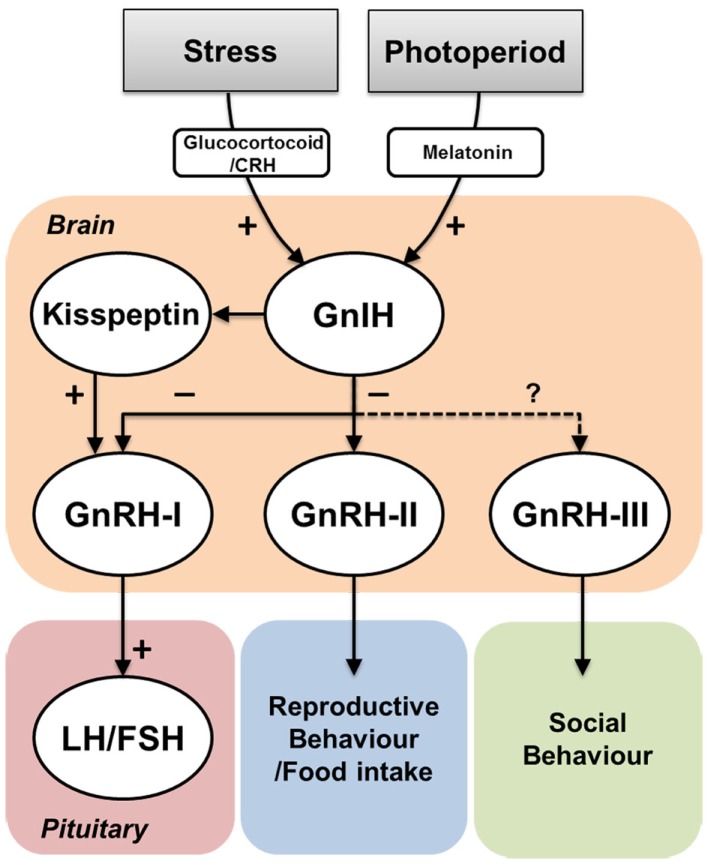
**Effect of environmental actions on GnIH system and its potential functions**. Environmental cues such as social stress or seasonal/diurnal change influence on GnIH neurons via hormonal mediators such as corticosterone or melatonin. GnIH neurons negatively act on GnRH-I and GnRH-II neurons, which influence on gonadotropin (LH and FSH) secretion in the pituitary and reproductive and/or food intake behaviors, respectively. In jawless and jawed fish, GnIH neurons send projection to GnRH-III neurons, which may regulate social behaviors. In mammals, GnIH neurons are also closely associated with kisspeptin neurons. However, the role of GnIH in kisspeptin neurons remains unknown.

GnIH is an inhibitory hypothalamic RFamide neuropeptide that has been characterized in various vertebrates including in the fish species (10, 14, 34, 52, 54). GnIH fibers and GnIH receptors are widely distributed in the brain as well as in the pituitary to regulate gonadotropin release (10, 34, 59, 81). GnIH fibers are also seen in close association with cells expressing other reproductive neuropeptides such as GnRH and kisspeptin neurons. GnIH and GnIH receptor signaling is also involved in several reproductive and non-reproductive functions, such as socio-sexual behaviors, appetite regulation, and stress response. Although the structure and function of the GnIH system is highly conserved in birds, mammals, and non-mammalian vertebrate species (Figure 4), there are still several questions that remain to be addressed in the case of fish GnIH because fish utilize a variety of reproductive strategies (140). For example, since the fish pituitary lacks the portal system of the ME and it is directly innervated by neurosecretory fibers (141), it would be interesting to know how GnIH acts on gonadotropes, whether directly or indirectly via other hypophysiotropic neurons such as GnRH neurons or the pineal gland. To understand the functional and physiological significance of vertebrate GnIH, further studies of GnIH system in a variety of vertebrates in particular in fish species would be very important.

## Author Contributions

Satoshi Ogawa wrote the paper. Ishwar S. Parhar edited the paper.

## Conflict of Interest Statement

The authors declare that the research was conducted in the absence of any commercial or financial relationships that could be construed as a potential conflict of interest.
